# Healthcare professionals’ views on access to vaccines in Nigeria: A cross sectional study

**DOI:** 10.1016/j.jvacx.2022.100235

**Published:** 2022-10-29

**Authors:** Obi Peter Adigwe, Davidson Oturu, Godspower Onavbavba

**Affiliations:** a National Institute for Pharmaceutical Research and Development, Plot 942, Cadastral Zone C16, Idu Industrial District, Abuja, FCT, Nigeria; b Aelex, 4th Floor, Adamawa Plaza, Plot 1099, 1st Avenue, Off Shehu Shagari Way, Central Business District, FCT, Abuja, Nigeria

**Keywords:** Pharmaceutical sector, Medicines’ Security, Vaccines, Public health, Manufacturing, Policy

## Abstract

Vaccines are important public health interventions that are critical in preventing the spread of infectious diseases. Sustainable access to these products is therefore critical in articulating contextual policies and strategies. This study aimed at exploring the views of healthcare professionals regarding perceived challenges and strategies that influence access to vaccines in Nigeria. A cross sectional study was undertaken amongst healthcare practitioners that attended a conference targeted at improving access to vaccines. A questionnaire was used for data collection, and analysis was undertaken using Statistical Package for Social Sciences version 25. Questionnaires were administered to a total of 604 participants, response rate was 87.1%, with male participants (54.4%) being slightly better represented than females (45.6%). A tenth of the participants (10.6%) were educated up to doctorate degree level, and a considerable proportion of the participants (43.6%) worked in the government sector. Slightly above three quarters (78.3%) of the participants were of the view that lack of local production capacity was an obstacle preventing access to vaccines, whilst above two thirds of the respondents (70.5%) were of the opinion that the current funding for research and development towards vaccines was sub-optimal. A total of 70.1% of the sample disagreed that the current policy environment was favourable to development of vaccines, whilst more than half of the participants (56%) perceived a lack of support by philanthropists and relevant foundations, for vaccines development in Nigeria. A majority of the participants (73.7%) indicated that sustainable access to vaccines in Nigeria could be achieved by harnessing local research capacity. This study identified critical challenges limiting access to vaccines in Nigeria and can consequently underpin relevant policy and practice reforms that aim to improve access to this public health tool.

## Introduction

Vaccines are biological products that can provide acquired immunity to a disease by inducing an immune response. This group of pharmaceuticals represent an important public health intervention that has a significant impact on infectious disease reduction globally [1]. Immunisation with effective vaccines brings immense value to families and communities by way of reducing the spread of infectious illnesses [2], [3]. Over the years, public health strategies underpinned by vaccination, have contributed to the control of common child killer diseases such as measles, diphtheria, and diarrhoea cases due to rotavirus [4]. Whilst significant progress has been made towards controlling and eradicating vaccine preventable diseases, substantial gaps still exist in terms of access to vaccines. Available data suggests that immunisation coverage in African region is decreasing [5]. Also, it was reported that a quarter of children from middle income countries in Africa did not receive their third dose of DPT vaccine in 2016, and average immunisation coverage dropped from 83% in 2015 to 77% in 2017 [6].

According to World Health Organisation, it is expected that all communities have unhindered access to essential medicines and vaccines [7]. However, this is not the case in Nigeria and other developing countries, where lack of Medicines’ Security is a major challenge to healthcare delivery. Improving local research capacity has been identified as one important strategy to improve access to vaccines [8]. This approach can also help ensure the achievement of local production as well as reduce dependence on importation of pharmaceutical products, especially those for which local firms have the capacity to produce [9].

Access to new and effective therapeutics have long been a challenge for Nigeria and other African countries. Manufacturing of pharmaceutical products by developed countries, is frequently geared towards profitability, leaving African nations behind [10]. Available evidence suggests Africa accounts for only 3% of global pharmaceutical production, and depending on the setting, between 70% and 90% of pharmaceuticals consumed on the continent are imported [11]. Following the COVID-19 pandemic, Nigeria started addressing Medicines’ Security challenges by providing loans to local pharmaceutical firms to support manufacturing [12]. However, local production of vaccines still remains a critical challenge in the country, thereby leaving the nation to rely on importation of all vaccines consumed in this setting. Considering the fact that healthcare professionals are important stakeholders when it comes to utilisation of vaccines and other therapeutics, it is important to assess their views on access regarding these critical health commodities. This study therefore aimed at assessing views of healthcare professionals on challenges and strategies towards improving access to vaccines in Nigeria.

## Methods

A cross sectional design was adopted for this study. The participants of the study were healthcare professionals that participated in a conference on improving access to vaccines in Nigeria and other developing countries, held in June 2021. The conference was hosted in Abuja, which is located in the Federal Capital Territory; the capital of Nigeria. The healthcare professionals comprise those in private sector, public sector, and development agencies. These practitioners were made up of medical doctors, pharmacists, nurses and other healthcare personnel practicing in different settings including hospitals, research organisations and pharmaceutical industries. The design of the questionnaire for the study was undertaken following an extensive literature review [13], [14], [15] and items in it include demography as well as aspects relating to challenges, policies and practices regarding access to vaccines. These thematic areas were focused on the aspects of vaccines manufacturing and research & development. A Likert type scale of 1 to 5 was used to assess the views of participants and the coding pattern was as follows: 1 = strongly disagree, 2 = disagree, 3 = neutral, 4 = agree, and 5 = strongly agree. The research instrument was validated by a team of experts with relevant research experience in this area, following which, a pilot testing of the questionnaire was undertaken by administration to 25 participants. The feedback received from the pretesting did not necessitate any major change. A convenience sampling strategy was adopted for data collection. Paper questionnaires were administered to all participants physically present, whilst an online questionnaire was generated using Google Forms and emailed to those that took part in the conference remotely. A reminder was sent to online participants so as to increase the response rate.

Ethical approval was obtained from the Health Research Ethics Committee of National Institute for Pharmaceutical Research and Development prior to the commencement of data collection. Informed consent was obtained from respondents that participated physically before administering questionnaires to them. Whilst for online participants, providing informed consent was a requirement to move to the questionnaire filling stage. All information provided was anonymised so as to maintain absolute confidentiality.

Following retrieval of questionnaires, online responses were downloaded in Excel format before extraction into Statistical Package for Social Sciences (SPSS) version 25. Paper-based responses were loaded directly into the software. Descriptive statistical analysis was carried out, and association of responses with socio-demographic characteristics was undertaken using chi square test. A *p* value of 0.05 or less represented the threshold for statistical significance.

## Results

### Demography

The questionnaire was administered to a total of 604 participants, and 526 of them responded by completing and returning it, giving a response rate of 87.1%. The final percentages presented excluded missing data. Male participants (54.4%) were slightly higher than females (45.6%), and participants who were above 60 years represented the least proportion. Further details about socio demographic characteristics are presented in Table 1.

**Table 1:** Socio demographic characteristics of participants.

**Variable**	**Frequency (%)**
Gender	
Male	286 (54.4)
Female	240 (45.6)
Age (years)	
≤ 30	145 (27.6)
31 – 40	162 (30.8)
41 – 50	91 (17.3)
51 – 60	108 (20.5)
Above 60	20 (3.8)
Highest Educational Level	
National Diploma/NCE	72 (13.9)
First Degree/HND	285 (55.0)
Masters’ Degree	106 (20.5)
PhD	55 (10.6)
Occupation	
Government Sector	223 (43.6)
Private Sector	204 (39.8)
Development Agency	45 (8.8)
Retired	18 (3.5)
Others	22 (4.3)

Note: HND = Higher National Diploma, NCE = National Certificate of Education.

## Challenges preventing access to vaccines

**Fig. 1:**
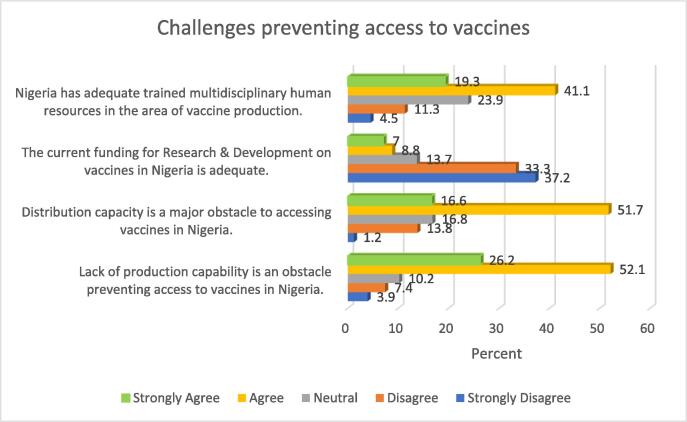
Challenges preventing access to vaccines.

Findings presented in Fig. 1 show that about two thirds of the study participants (60.4%) indicated that there were adequate trained multidisciplinary human resources in the area of vaccine production, whilst 15.8% indicated otherwise. Close to three quarters of the sample (70.5%) were of the view that the current funding for research and development on vaccines was inadequate.

Also, from Fig. 1, two thirds of the study participants (68.3%) were of the opinion that distribution capacity was a critical factor preventing access to vaccines, whilst more than three quarters of the respondents (78.3%) agreed that a lack of local manufacturing capacity contributed to sub-optimal vaccines’ access in Nigeria.

### Policies and practices on vaccines

**Fig. 2:**
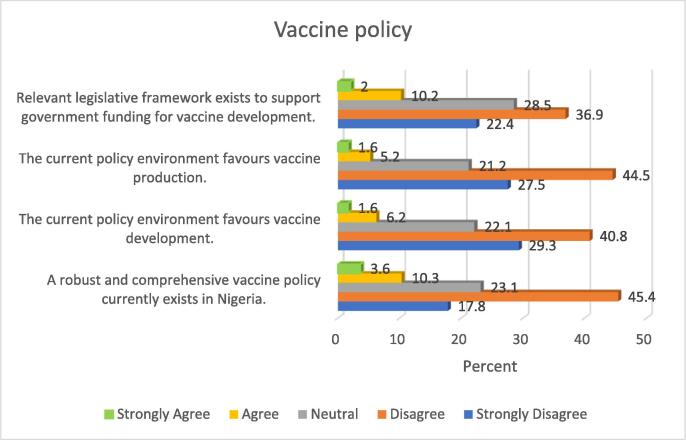
Policies on vaccines.

Majority of the participants (59.3%) disagreed that relevant legislative framework exists to support government funding for vaccines development, whilst close to three quarters of the respondents (72%) indicated that the current policy environment does not favour vaccine production. Other relevant details on vaccine policy are presented in Fig. 2.

Further details presented in Fig. 2 revealed that two thirds of the study cohort (63.2%) disagreed that a robust and comprehensive vaccine policy currently exists in Nigeria.

**Fig. 3:**
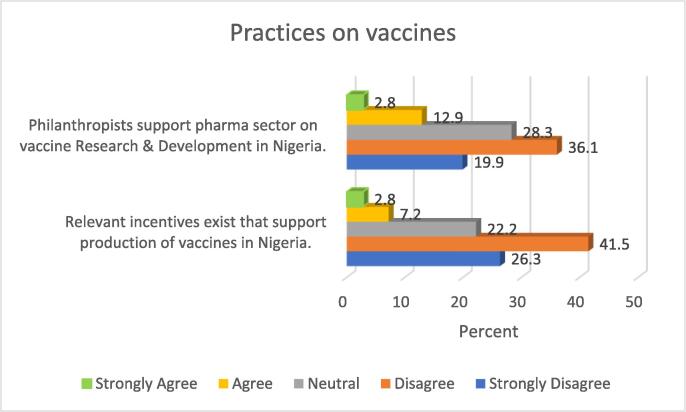
Practices on vaccines.

Majority of the participants (56%) in the study disagreed that the support from philanthropists towards vaccine research and development activities had been optimal. Conversely, only 15.7% of the sample seemed to agree with the contrary position. Other relevant details are presented in Fig. 3.

Also, as presented in Fig. 3, two thirds of the study participants (67.8%) disagreed that relevant incentives exist that support manufacturing of vaccines in the Nigerian setting.

### Strategies to improve access to vaccines

**Fig. 4:**
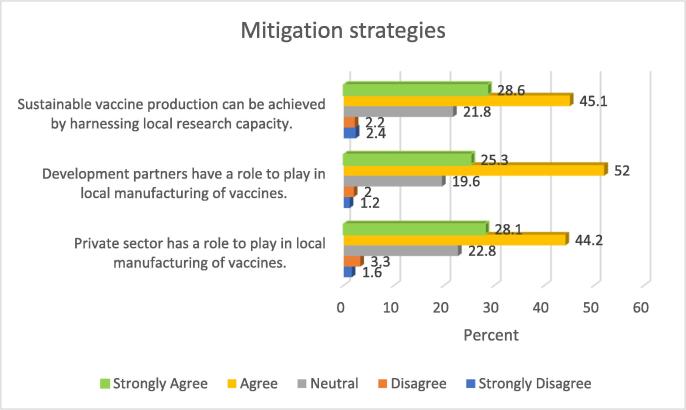
Strategies to improve access to vaccines.

About three quarters of the sample (73.7%) were of the view that sustainable manufacturing of vaccines can be achieved by harnessing local research capacity, whilst slightly above this proportion (77.3%) were of the opinion that the role of development partners was critical in stimulating local manufacturing. Further details are provided in Fig. 4.

Close to three quarters of the respondents (72.3%) in this study were of the view that private sector has a role to play in local manufacturing of vaccines.

In addition to the descriptive statistics, inferential statistical analysis was undertaken to determine association between socio-demographic characteristics and some of the variables. Findings showed that more of the participants with doctorate degrees (85.2%) were of the opinion that the current policy environment does not favour vaccine development compared to those with masters’ degrees (78.6%), first degrees (64%), and participants with diplomas (67.1%). This finding was statistically significant (*p* < 0.001). Similarly, the more educated participants as indicated by their doctorate degrees, were of the view that the current funding for research and development on vaccines was inadequate (*p* = 0.001). Further details are provided in Table 2.

**Table 2:** Association between level of education and views of participants.

**Statement**	**Educational Level**	**Strongly Disagree n (%)**	**Disagree n (%)**	**Neutral n (%)**	**Agree n (%)**	**Strongly Agree n (%)**	**X^2^**	***P-value***
The current policy environment favours vaccine development in Nigeria.	ND/NCE	35 (50.0)	12 (17.1)	18 (25.7)	5 (7.1)	0 (0.0)	45.516	<0.001
	First degree/HND	63 (23.6)	108 (40.4)	65 (24.3)	23 (8.6)	8 (3.0)		
	Master’s	31 (30.1)	50 (48.5)	19 (18.4)	3 (2.9)	0 (0.0)		
	PhD	15 (27.8)	31 (57.4)	8 (14.8)	0 (0.0)	0 (0.0)		
The current policy environment favours vaccine production in Nigeria.	ND/NCE	32 (45.7)	19 (27.1)	13 (18.6)	6 (8.6)	0 (0.0)	39.139	<0.001
	First degree/HND	61 (23.0)	112 (42.3)	65 (24.5)	19 (7.2)	8 (3.0)		
	Master’s	28 (27.2)	58 (56.3)	16 (15.5)	1 (1.0)	0 (0.0)		
	PhD	14 (26.4)	28 (52.8)	11 (20.8)	0 (0.0)	0 (0.0)		
The current funding for Research & Development on vaccines in Nigeria is adequate.	ND/NCE	18 (25.7)	24 (34.3)	19 (27.1)	8 (11.4)	1 (1.4)	33.883	0.001
	First degree/HND	91 (33.2)	95 (34.7)	38 (13.9)	27 (9.9)	23 (8.4)		
	Master’s	51 (48.6)	30 (28.6)	10 (9.5)	6 (5.7)	8 (7.6)		
	PhD	27 (50.0)	20 (37.0)	1 (1.9)	3 (5.6)	3 (5.6)		

## Discussion

A number of novel insights relating to access to vaccines in Nigeria emerged from this study. Majority of the participants were of the view that the inability of the Nation to manufacture vaccines contributed to its people’s lack of access to vaccines. This finding validates the Medicines’ Security concept which argues that unless a people exert sufficient control over how their pharmaceuticals and other healthcare commodities are produced, it will be difficult to guarantee sustainable access to affordable high quality products in that setting [16], [9]. Apart from improving access to high quality products, local manufacturing of vaccines can significantly boost socioeconomic development, which include job creation, knowledge transfer, as well as revenue generation [17]. Currently, all vaccines used in Nigeria are imported, and this is a contravention of the National Drug Policy which was designed to satisfy up to 70% of national pharmaceuticals’ needs through local manufacturing [18]. The COVID-19 pandemic has however emphasised the criticality of local manufacturing of vaccines, and this was highlighted by the high rate of COVID-19 vaccine nationalism which disenfranchised developing countries such as Nigeria [19].

In this study, close to three quarters of the participants disagreed with the adequacy of funding for research and development with respect to vaccines as they were of the opinion that financial resources in this area were not commensurate to the contextual needs. This suggests that there is a desperate need to prioritise research in this critical area, due to the criticality of vaccines as public health tools for preventing, controlling and mitigating infectious disease spread [20], [21]. More than three quarters of the participants indicated that distribution capacity was an obstacle preventing access to vaccines, thereby validating previous findings that had identified gaps in the area of cold chain and supply chain of vaccines in Nigeria [22], [23], [24], [25]. Conversely, majority of the participants believed that there was enough human resource capacity in this area, and this is at variance with previous reports [26], [27], [28]. With respect to policy, majority of the participants disagreed that the current policy environment favoured vaccine development and manufacturing. They also opined that there was a lack of relevant legislative framework to support government's prioritisation and funding of vaccine development, suggesting the need for relevant policy and legislative reforms to stimulate sustainable progress in this area. Furthermore, from the findings in this study, it emerged that healthcare professionals perceived a lack of partnership and support from development partners and philanthropists. This finding about the Nigerian setting is at variance with the practice in developed countries where up to 40 percent of similar health research are funded through partnership with charitable organisations [29].

Two thirds of the sample in this study were of the view that there was a paucity of relevant incentives that support vaccines’ manufacturing in the Nigerian setting. The empirical evidence from this study means that government needs to review critical aspects of existing policies and legislation governing the entire vaccines value chain. Fiscal and industrial technical tools to be considered in this regard include specialised industrial zones, waivers and tax breaks, grants and dedicated low interest, long tenured loans. This strategy has been found effective in stimulating innovation, increasing research output, as well as promoting manufacturing of products that may not be immediately economically viable, but are nevertheless critical in saving lives and achieving broader long term socioeconomic goals [30]. Participants in this study also supported harnessing of local research capacity, as strategy for sustainable access to vaccines in Nigeria, and this is similar to previous findings in the Nigerian setting [9]. In addition, respondents strongly indicated that the private sector and development partners have important roles to play in achieving local manufacturing of vaccines. This implies the urgent and critical need for a comprehensive, robust and all-inclusive engagement plan that links all stakeholders including research entities, government, development partners and the private sector. These are the sort of collaborative strategies that would yield sector wide development and consequently underpin sustainable access to vaccines in Nigeria.

Although the study design aimed at comprehensively capturing industry views, limitations may arise due to the broad-based selection of healthcare professionals irrespective of their role in the vaccine value chain. This is however the first study in this setting to comprehensively assess the views of different healthcare practitioners on policies and practices relating to access to vaccines. Further studies are therefore recommended to explore these critical concepts as well as to deepen the emergent findings that emanated from this study, especially as they concern each practitioner category of the vaccine development value chain.

## Conclusion

This study has provided insights from the perspectives of healthcare professionals on some of the challenges preventing access to vaccines as well as important strategies to improve access to these healthcare commodities. It emerged that lack of local production of vaccines and inadequate funding of research and development activities in the field, have contributed significantly to a lack of access to this important public health tool in Nigeria. Also, the current policy environment was reported as unfavourable for development and manufacturing of vaccines, suggesting the need for urgent reforms. Furthermore, harnessing local research capacity was identified as an important strategic initiative that can stimulate sustainable local production of vaccines.

To improve access to vaccines in Nigeria, urgent policy and practice reforms led by government are required. Involvement of key stakeholders such as philanthropists, the private sector and development partners will ensure that the process is sustainable and resource efficient. Prioritisation and optimal funding of the pharmaceutical sector will catalyse vaccines’ research and development in Nigeria. Similarly, reviewing existing incentive framework for local manufacturers can also stimulate interest in the production of this critical public health tool.

## Declaration of Competing Interest

The authors declare that they have no known competing financial interests or personal relationships that could have appeared to influence the work reported in this paper.

## Data Availability

Data will be made available on request.
